# Part 2. Comparison of emergency washing solutions in 70% hydrofluoric acid-burned human skin in an established *ex vivo* explants model

**DOI:** 10.3109/15569527.2010.534748

**Published:** 2010-11-18

**Authors:** François Burgher, Laurence Mathieu, Elian Lati, Philippe Gasser, Laurent Peno-Mazzarino, Joël Blomet, Alan H Hall, Howard I Maibach

**Affiliations:** 1 Scientific Action Group, Prevor Laboratory, Valmondois, France; 2 BIO-EC Laboratory, Longjumeau, France; 3 Toxicology Consulting and Medical Translating Services, Inc., Laramie, WY, USA and Colorado School of Public Health, Denver, CO, USA; 4 Department of Dermatology, University of California-San Francisco, San Francisco, CA, USA

**Keywords:** Human skin explants model, decontamination, hydrofluoric acid skin decontamination, hydrofluoric acid burns, Hexafluorine®

## Abstract

*Background:* Hydrofluoric acid (HF) is a small and partially dissociated acid (p*K*_a_ 3.2), able to deeply penetrate into human skin in addition to the corrosiveness of the hydrogen ion (H^+^) and the toxicity of the fluoride ion (F^-^). However, there has been a lack of experimental studies to objectively characterize the results of human HF skin exposure decontamination.

*Methodology/principal findings:* A previously established experimental method using a human skin explants *ex vivo* model (Part 1. Experimental 70% hydrofluoric acid (HF) burns: Histological observations in an established human skin explants ex vivo model) described the lesions that appeared following 70% HF penetration. Within 5min, 70% HF penetrates to the dermis. Using the same experimental conditions, a comparison study of two different washing protocols was performed: water + topical calcium gluconate (CaG) versus Hexafluorine®. In these conditions, washing for 15min with running tap water followed by topical CaG ointment only delayed burn onset, while severe tissue damage appeared later. In contrast, after washing with Hexafluorine® over 10 min, no histological lesions developed. These results are in accordance with the results of accidental human industrial case reports.

*Conclusion/significance:* Amphoteric and hypertonic Hexafluorine® can deactivate H^+^ and chelate F^-^ ions. Based on these results, it should be considered as a promising first-aid decontamination solution to prevent or minimize significant local and systemic consequences of concentrated HF skin exposures.

## Introduction

Hydrofluoric acid (HF) is widely used for the manufacture of fluorinated organic compounds, inorganic fluorides, uranium treatment, fluorocarbon products, and fluoropolymers and derivatives. HF is also used in the metal industry for scouring and shining steels (especially for stainless steel products) and other metals (such as for pickling titanium), in the oil refinery industry (catalyst for alkylation), in electronics (for the surface treatment of semiconductor components), in the glass industry (for engraving, etching, frosting, and polishing of glass and crystal, and for quartz purification), in construction (for facade cleaning), in the chemical industry (particularly in laboratories), in the refrigeration industry, and in the photovoltaic industry (polysilicones, fluoropolymer films). Dilute HF (usually 6-13%) has household uses such as rust stain removal, aluminum cleaning and polishing (for automobile wheels), and for colored wood cleaning.

The worldwide production of HF is increasing with > 1 million tons per year (including US production capacity of about 434,000 tons in 2002 [1] plus 480,000 tons imported [2] and Chinese production capacity of about 870,000 tons in 2008 [3]).

HF is a particularly dangerous acid due to its two properties: corrosiveness and toxicity. In addition, as it is only partially dissociated (pK_a_ = 3.2), this small molecule is capable of penetrating deeply into tissues.

HF is an acid that induces severe tissue necrosis and is a serious systemic poison due to two mechanisms of ion delivery: a corrosive hydrogen ion (H^+^) associated with cutaneous and mucous membrane lesions [4] and with ocular [5] and respiratory tract [6-8] damage; a toxic fluoride ion (F∼) with slower onset local and systemic effects (especially decreased myocardial contractility and cardiac arrhythmias such as tachycardia, torsades de pointes ventricular tachycardia, and ventricular fibrillation [9]), with potentially lethal effects [10-14].

The F^-^ ion chelates calcium and magnesium [15] forming insoluble salts: CaF_2_ and MgF_2_. Soluble salts such as NaF and KF are also produced and act at high concentrations as direct cellular poisons.

This chelation induces metabolic disorders [16,17] that lead to delayed cellular death and secondary tissue necrosis. Moreover, the binding of calcium is thought to increase cell membrane permeability to potassium, resulting in neuronal depolarization and intense pain [18,19]. Fluoride ion is also known to attack enzymes and cell membranes [20].

The systemic consequences of HF are due to increased fluoride concentrations in the body, which can modify the blood levels of calcium (hypocalcemia), magnesium (hypomagnesemia), and potassium (hyperkalemia) [21-23]. HF in concentrations of 50% or greater (including anhydrous hydrogen fluoride) causes immediate, severe, throbbing pain, and a whitish discoloration of the skin, which usually forms blisters. HF concentrations of 20-50% may produce pain and swelling, which may be delayed in onset for up to 8 h. HF solutions of <20% cause almost no immediate pain but may cause delayed serious skin injury 12-24h later [24] (Table 1). Especially in the case of prolonged exposure to very dilute solutions, symptoms may even be delayed in onset for several days.

**Table 1:** Pain and skin lesions: depending on HF concentration.

Pain and Skin lesions	Concentration of HF
Immediate pain and rapid necrosis	50% or greater
Delayed pain and burn 1.8 h	20.50%
Delayed pain and necrosis until 24 h	≤20%

The systemic toxicity is potentially life-threatening [25] depending on the available total amount of free fluoride ions that penetrate into the body. Lethal risk is correlated with the HF concentration, the total body surface area (TBSA) exposed, and the duration of contact [26].

### Study objectives

This study was performed to demonstrate the extent of epidermal and dermal lesions following contact with 70% HF on living human skin explants *ex vivo* and to compare the efficacy of different decontamination washing protocols.

Observation of the general morphology allowed characterization of the nature and extent of 70% HF-induced skin lesions at the tissue and cellular levels over time following exposure alone or exposure followed by decontamination.

Comparison of two different washing protocols was done: running tap water followed by a one-time calcium gluconate (CaG) gel topical application, and Hexafluorine® (a specific *active* HF decontamination solution).

## Methods

The study was performed at BIO-EC Laboratories, Longjumeau, France in accordance with a previously described and established experimental burn model using human skin explants *ex vivo* (see Part 1). Tested products:

1. 70% HF (FLUKA Ref. 47610, batch 7125A, with an exactly titrated concentration of 73.0%)
2. Running tap water
3. CaG gel with topical application of 1 mg/cm^2^ (KAYS MEDICAL, Lot F022011/09, 2.5% gel)
4. Hexafluorine® spray (miniDAP 200 mL, batch 971201C, Prevor Laboratory, Valmondois, France).

### Explants preparation

The 70 human skin explants utilized were prepared from donated skin from an elective abdominoplasty from a 35-year-old woman (Reference P556). Verbal-informed consent was obtained (see Part 1 for details of the informed consent procedure). The diameter of each explant was ∼10mm. The study was performed in triplicate to insure the internal consistency of the method, not for statistical purposes. The explants were preserved alive in BEM medium (BIO-ECs Explant Medium batch No. 071107) at 37°C in a moist atmosphere containing 5% CO_2_ for 12-15 hours before the study began. These explants were divided into four groups (Table 2).

**Table 2:** Explants and treatment groups.

Group	Number of Explants	Treatment	Histological sampling Times
T	20	None	T0, 20 s, 5, 10, 15, 30 min, 1, 2, 4 and 24 h
F	18	30 μL (47 μL/cm^2^) 70% hydrofluoric acid exposure without washing	20 s, 5, 10, 15, 30 min, 1, 2, 4, and 24 h
FWCaG	16	30 μL (47 μL/cm^2^) 70% hydrofluoric acid applied for 20 s, then washing with water + calcium gluconate topically	After the end of washing at: 5, 10, 15, 30 min, 1, 2, 4, and 24 h
FHexa	16	30 μL (47 μL/cm^2^) 70% hydrofluoric acid applied for 20 s, then washing with Hexafluorine® (2 washes with 200 mL each per explant)	After the end of washing at: 5, 10, 15, 30 min, 1, 2, 4, and 24 h

In all treatment groups, 70% HF saturated filter paper disks were left in contact for 20 s and then removed.

### Application of products and washing solutions

The HF solution was applied topically to the explants of groups F (70% HF; no washing), FWCaG (70% HF; water washing + topical CaG); and FHexa (70% HF; Hexafluorine® washing) by deposition on filter paper disks (9 mm diameter; MEDIAS FILTRANS DURIEUX S.A., *papier filtre* No. 268) previously saturated with 30 μL (47 μL/cm^2^) of 70% HF solution.

For group F, HF was left in contact with the skin for 20 sec and then the filter paper disks were removed. No washing was done. After the HF-saturated disks were removed, sampling for histological observations was done at 5, 10, 15, and 30min, 1, 2, and 4h, and 24h.

For groups FWCaG and FHexa: after 20 sec of contact, the disks were removed. Then the different washing protocols were performed. The explants of group FWCaG were washedwith runningtap water for 15 min (∼2000 mL per explant), and then topical CaG gel (2.5%) was applied, one time only, to each explant at a dose of 1 mg/cm^2^. The explants of group FHexa were washedwith Hexafluorine® applied as a spray of 400 mL over ∼10 min. Group T (negative controls) did not receive any treatment. The total observation time ranged from 20 sec to 24 h.

### Histology sampling

Group T (negative controls): At the beginning of the study (T0), two explants of the control group were sampled and fixed in Bouin's solution. Two other explants were sampled and similarly fixed at each sampling time: 20 sec, 5, 10, 15, and 30min, 1, 2, and 4h, and 24h.

Group F (positive controls): Twenty seconds after the beginning of the 70% HF application, two explants from group F were sampled and fixed immediately after removal of the saturated disk. Then, two explants were sampled and fixed in the same manner at each of the sampling times: 5, 10, 15, and 30min, 1, 2, and 4h, and 24 h.

Groups FWCaG and FHexa: Five minutes after the washing step, two explants from each of groups FWCaG and FHexa were sampled and fixed in Bouin's solution. Then two explants from each group were sampled and fixed in the same manner at each of the sampling times: 10, 15, and 30min, 1, 2, and 4h, and 24h.

### Histology

Tissue fixation, sample preparation, and histology procedures are described in detail in Part 1.

Alterations of cellular structures were searched for during penetration of HF through the main skin layers: upper epidermis (stratum corneum + lucidum + granulosum), basal epidermis (stratum germinativum), and papillary and reticular dermis.

## Results

Group T (no HF exposure; no washing of explants— negative controls): At T0, the skin showed four to five cellular sites with good morphology. In the papillary and the lower reticular dermis, the cellular structures had good morphology. Whatever the time of survival, no modifications of the epidermal and dermal structures were observed up to 24 h. (Please refer to Part 1, Figure 2 for the histological appearance of normal control explant skin.)

**Figure 1:**
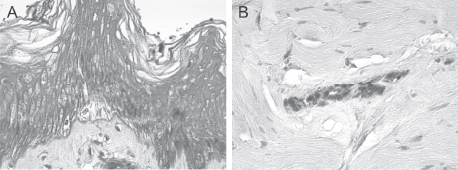
(A) and (B) Exposed 70% HF, nonwashed explants, aspect at 24 h after a 20-sec exposure. Epidermis (A) and dermis (B). At 24h after a 20-sec exposure, the epidermis presented completely necrotic structures with the appearance of coagulation necrosis. The lesions were less intense in the papillary and reticular dermis (See colour version of this figure online at www.informahealthcare.com/cot)

Group F (explants exposed to 70% HF for 20 sec on filter paper disks, which were then removed; no washing— positive controls)

After 20 sec of contact, no deterioration of the structures of either the epidermis or dermis was observed. At 5 min after a 20-sec exposure, very clear cellular deterioration was observed in the epidermis and papillary and reticular dermis. Lesions clearly increased at 10, 15, and 30 min and 1 h after a 20-sec exposure, with the marked appearance of coagulation necrosis including acido-philic cytoplasm and pyknotic nuclei throughout all the skin layers. At 24h after a 20-sec exposure, the epidermis presented completely necrotic structures. The lesions were less intense in the papillary and reticular dermis (Figure 1A and 1B).

**Figure 2:**
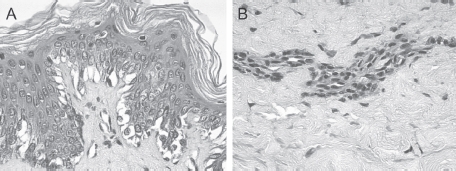
(A) and (B) HF-exposed explants rinsed with water + CaG, aspect at 24h after a 20-sec exposure. Epidermis (A) and dermis (B). Edematous changes were visible in the epidermal basal layer at 24 h after a 20-sec exposure. An appearance of coagulation necrosis including pyknotic nuclei and acidophilic cytoplasm was noted in the papillary dermis. These changes were less apparent in the reticular dermis. (See colour version of this figure online at www.informahealthcare.com/cot)

Group FWCaG (explants exposed to 70% HF for 20 sec on filter paper disks, which were then removed and the explants were rinsed with running tap water followed by one topical application of 2.5% CaG gel).

At 5 and 10 min after a 20-sec exposure, no deterioration of the structures of either the epidermis or dermis was observed.

At 15 min after a 20-sec exposure, very clear cellular deteriorations appeared in the epidermis and in the papillary and reticular dermis. These were decreased at 30 min, and were no longer observed after 1 and 2h after a 20-sec exposure. Slight edematous changes were visible in the epidermal basal layers at 4h and these were clearly increased at 24h after a 20-sec exposure (Figure 2A and 2B).

Group FHexa (explants exposed to 70% HF for 20 sec on filter paper disks, which were then removed and the explants washed with Hexafluorine® for ∼10 min (400 mL)).

**Figure 3:**
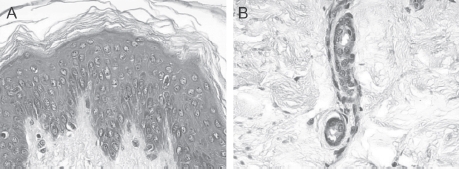
(A) and (B) HF-exposed explants rinsed with Hexafluorine®; aspect at 24h after a 20-sec exposure. Epidermis (A) and dermis (B). No deterioration of the structures of either the epidermis or dermis was observed. (See colour version of this figure online at www.informahealthcare.com/cot)

At 5, 10, 15, and 30 min and 1, 2, 4, and 24 h after a 20-sec exposure, no deterioration of the structures of either the epidermis or dermis was observed. Washing by spraying Hexafluorine® for ∼10 min (400 mL) gave a homogeneous result on the explants. Whatever the time of observation, no burn lesions were observed (Figure 3A and 3B).

Table 3 summarizes the results of the observations for all study groups and for each of the three main skin explant layers: epidermis, papillary dermis, and reticular dermis. It makes it possible to follow the evolution of the tissue lesions following a 20-sec cutaneous exposure to 70% HF and, indirectly, to make a correlation with the overall kinetics of HF penetration through human skin explants layers. It also allows comparison of human skin explants exposed to 70% HF and either not washed or washed with different washing protocols. As can be appreciated, water washing followed by application of topical CaG gel did delay the onset of burn lesions and mitigated their severity compared with positive controls (70% HF; no washing), when the explants were washed with Hexafluorine®, no burns developed and there were no differences in the histological appearance from those of the untreated negative control group.

**Table 3:** Schematic summary of the histological results for all the groups and for each skin layer.

Time of exposure and skin layers		T (Control untreated group) 20 explants	F (HF without washing) 18 explants	FWCaG (HF + water washing + calcium gluconate) 16 explants	FHexa (HF + Hexafluorine® 400 mL) 16 explants
T0	Epidermis	GM = good morphology	GM	GM	GM
	Papillary dermis				
	Reticular dermis				
20 s	Epidermis				
	Papillary dermis				
	Reticular dermis				
5 min	Epidermis		PN + AC		
	Papillary dermis				
	Reticular dermis		PN + CA moderately		
10 min	Epidermis		PN= pyknotic nucleiAC = acidophilic cytoplasm		
	Papillary dermis				
	Reticular dermis				
15 min	Epidermis			PN + AC moderately	
	Papillary dermis			PN + AC	
	Reticular dermis				
30 min	Epidermis			Some necrotic cells	
	Papillary dermis			GM	
	Reticular dermis				
1 h	Epidermis				
	Papillary dermis				
	Reticular dermis				
2 h	Epidermis				
	Papillary dermis				
	Reticular dermis				
4 h	Epidermis			Slightly edematous cells with mild acantholysis	
	Papillary dermis			GM	
	Reticular dermis				
24 h	Epidermis		Totally necrotic	Very edematous cells with a very clear cytoplasm	
	Papillary dermis		PN + AC	PN + AC	
	Reticular dermis			Lesser alterations	

GM, good morphology; PN, pyknotic nucleus; AC, acidophilic cytoplasm.

PN and AC are described as part of the appearance of coagulation necrosis.

In all treatment groups, 70% HF saturated filter paper disks were left in contact for 20 s and then removed.

## Discussion

HF burns have specific pathophysiological mechanisms that require specialized treatment to prevent serious sequelae. Appropriate urgent first aid and secondary medical management can improve the prognosis. Initial decontamination must be efficacious to avoid or minimize the extent (TBSA) and depth of HF burns and to prevent systemic absorption and distribution of the F∼ ions responsible for potential life-threatening systemic toxicity [27]. Efficacious washing must attempt to arrest the progression of injury, but obviously cannot reverse tissue necrosis that has already occurred.

Up to now (see Part 1), there has been a lack of histological studies allowing real-time observation of cellular damage during penetration of concentrated HF into human skin, as well as real-time observation of how different decontamination measures may affect such penetration and potential skin injury.

In a previous study (see Part 1), human skin explants exposed *ex vivo* to 70% HF were tested. It was shown with topical exposure to 30 μL (47 μL/cm^2^) of 70% HF, 20 sec of contact was sufficient to penetrate the upper layers of the skin explants. By 5min after a 20-sec 70% HF exposure, all the epidermal and dermal layers of the skin were damaged, but not totally and irreversibly destroyed.

In this study, it was not a question of the treatment of fully developedHF skinburns, as hasbeendone previously in a rat model [28]. Rather, it was a question of which initial decontamination measures are most likely to prevent or mitigate the effects of HF human skin exposure. Such initial measures using water washing are described in the ANSI/ISEA Z358.1-2009 American National Standard for Emergency Eyewash and Shower Equipment. There are similar measures noted in the European Norms (European Committee for Standardization: NF EN 15154-3; June 06, 2009).

The present study was carried out in conditions such as to be as close as possible to potential exposure to high concentrations of HF likely to be encountered in industrial settings.

Explants from the control group remained alive during the 24 h of the experiment. Seventy percent HF penetrated human skin explants after 20 sec of exposure. Without decontamination washing, the first cellular alterations were noticeable by 1 min after a 20-sec exposure. The reticular dermis was reached and injured by 5 min after a 20-sec exposure. At 5 min after a 20-sec exposure, a massive attack of all the cutaneous layers was already present. The lesions then remained stable between 10 min and 4h. At 24 h after a 20-sec exposure, the epidermis was totally necrotic while the dermis was clearly altered (appearance of coagulation necrosis including pyknotic nuclei and totally acidophilic cytoplasm). Similar observations were reported by Ohtani et al. [29] in a human case report with 60% concentration HF lethal chemical burns.

In the past, water washing was a great advance in the first-aid care for victims of corrosive skin exposures. Historically, evidence for the seriousness of HF burns and the need for efficacious first-aid measures was initially described in France by Thenard and Gay Lussac [30] in the first quarter of the 19th century.

At this early date, these authors had already noted that the development of HF burns can sometimes be slowed and that the appearance of the lesions could be delayed in onset from 1 to 8 h after exposure. They also experienced the acute pain sensation which “*can avoid sleeping and inducefever”* [30]. Thenard and Gay Lussac experimented upon themselves and discovered that a dilute solution of potassium hydroxide was able to stop the development of HF burns and could alleviate the pain sensation due to its chemical activity against the corrosive effects of HF. For unknown reasons, this interest in the concept of *active* HF decontamination was lost until the mid-1960s.

At that time, *passive* decontamination with copious prolonged water washing was and has continued to be recommended. Using water washing immediately after skin exposure allows dilution and mechanical rinsing of the HF from the skin. However, water washing alone is not always sufficient for preventing or minimizing HF burn injuries, especially with exposure to high concentrations of HF and/or large TBSA exposures.

A number of case reports show that HF burn victims have required surgical treatments and that others have died within a short time after exposure to concentrated HF, despite immediate copious water washing. Fatalities have occurred with exposure to highly concentrated HF (i.e. anhydrous HF) with only a small TBSA exposure on thin and highly vascularized skin such as that on the face and in the genital area [31-34].

A more theoretical approach to HF decontamination reveals that the first few seconds to minutes of washing are critical to prevent or minimize the development of HF skin burns. Some authors have therefore suggested a shorter initial water washing duration [24,35,36]. Segal [24] suggests that the duration of water washing should be limited to 5min when a specially prepared iced ben-zalkonium chloride solution or CaG gel is available. The concept of an efficacious *active* initial washing for HF exposures is derived from this approach. Water has no active binding or chelating properties for any chemical substance and does not inactivate either of the skin tissue injury mechanisms of HF. As water is hypotonic, it cannot be expected to stop penetration of HF throughout all layers of the skin and may, in fact, enhance such penetration. Schrage et al. [37] have demonstrated that cellular damage following exposure to a corrosive substance is actually enhanced in healthy corneal cell cultures by hypo-osmolar washing with tap water. Faced with the limitations [38] of water washing and based on theoretical concepts of *active* decontamination, an approach that allows deactivation of both HF's corrosive and local toxic potential is logical.

Free fluoride ions play a key role in local tissue toxicity, as well as in systemic toxicity. Therefore, improvement of first-aid measures for HF exposures initially focused only on the F” ion. Studied and published protocol changes to improve the outcome of HF burns only considered measures to be done after water washing [39]. Very few such studies considered acid (H^+^) neutralization and F∼ chela-tion as the initial first-aid measures after HF exposure [40].

Topical CaG was first considered for binding F∼ ions and interfering with their chelation of tissue calcium. The first publication describing HF burn therapy with CaG dates from 1964 [41]. At the current time, CaG is the product most often used worldwide for secondary care of HF burns. Various application/administration protocols have been proposed. Repeated topical application by inunction of 2.5-5.0% CaG gel (massaging the gel into the burn site seems to increase skin penetration over a 24-h period) or continuous ointment application (such as inside an over-large surgical glove for finger or hand exposures). As the CaG-F ion chelation property maybe insufficient to reach F∼ ions that have already penetrated deeply into the skin tissues, subcutaneous injection has been proposed to improve efficacy [29]. The skin is infiltrated with CaG underneath the burned area and the immediately adjacent skin. The usual dose is limited to 0.5mL/cm^2^ skin surface area of a 5% or 10% CaG solution (with a maximum of 0.5 mL per digit for finger burns). Care must be taken not to use *calcium chloride* solution in this manner because it can itself cause significant local tissue necrosis and sloughing. Pain that is not relieved or recurs is a clinical indication for further CaG infiltration.

Both intravenous and intra-arterial infusion of CaG can be done to treat systemic toxicity or hand/foot burns. These exposure routes are indicated for systemic toxicity signs and symptoms, or for more extensive HF burns or burns not responding to topical or subcutaneous CaG treatment [21,34,42-45]. Either CaG or calcium chloride may be infused intravenously, but only CaG may be infused intra-arterially. An intravenous Beir-block technique may also be used for HF extremity burns [46]. Intra-arterial infusion of CaG can have adverse effects such as arterial spasm and local bleeding [47].

Williams et al. [48] showed that 2.5% CaG significantly reduced burn size as early as 1 h after a single application on the shaved hind legs of rats burned with 70% HF. Magnesium sulfate has also been recommended for topical application, infiltration, or infusion [49-53]. Some authors have proposed that CaG be combined with dimethyl sulfoxide (DMSO) as a solvent vehicle to improve the calcium penetration, especially for nail or nail bed exposures [25,54].

Algorithms for HF splash victim management were studied by Kirkpatrick et al. (1995) [54], using 2.5% CaG gel topically and oral CaG tablets before admission to hospital, and intravenous infusion of 20 mL of 10% CaG solution under medical supervision after hospital admission. Dünser et al. [26] have also provided concise recommendations for therapy of major HF burns. Ohata et al. [55] described seven cases in which relief of severe pain occurred with CaG therapy. Nguyen et al. [43] described an unusual case where carotid intra-arterial infusion of CaG was used successfully to treat facial HF burns [43].

In the present study, a 15-min tap water washing followed by a single topical application of 2.5% CaG gel in one intervention leg was utilized to be consistent with common currently recommended first-aid measures.

In the FWCaG group, after 20 sec of exposure to 70% HF and then washing with tap water for 15 min followed by a single topical application of 2.5% CaG gel, a temporary delay in the onset of the appearance of tissue lesions was observed. The histological effects only became visible after 15 min, in comparison with the exposed but not washed control group in which the explants showed evidence of damage in all tissue layers after 5 min. In addition, in the FWCaG group, there was a resumption of evolution of the tissue lesions after 4h. At 24 h (at the conclusion of the experiment), HF chemical lesions were clearly present, though not as severe in the exposed but not washed control group.

As the tissue lesions continued to evolve from 4 to 24 h, this provides support for recommendations for repeated application of topical CaG. As well, in some cases topical CaG may need to be supplemented or replaced with par-enteral CaG administration (subcutaneous infiltration, intravenous or intra-arterial injection, or use of a Bier block technique for extremity burns).

When the results in the FHexa group of explants (exposed to 70% HF for 20 sec and then washed for ∼10 min with 400 mL of Hexafluorine®), which developed no lesions at any sampling time, were compared with the unexposed and not washed control group (group T), there were no differences in histological appearance at any sampling time from 5 min to 24h after a 20-sec exposure. Compared with the FWCaG group (20 sec of 70% HF exposure, then 15min of tap water washing followed by a single topical application of 2.5% CaG gel), the Hexafluorine® group developed no lesions at any sampling time, washing with tap water followed by topical CaG only delayed the onset of the appearance of the HF lesions and decreased their severity. There results suggest that Hexafluorine® acts on both mechanisms of HF skin injury: corrosiveness and local toxicity. Hexafluorine® deactivates the corrosive H^+^ ion and chelates the toxic F^+^ ion of HF. Moreover, it can prevent or minimize the penetration of undissociated HF into the tissues because it is hypertonic and establishes an osmotic gradient away from the skin surface.

The experimental results presented here are in accordance with published clinical results regarding the use of Hexafluorine® in over 30 cases of HF splashes in industrial settings [56,57] where no severe burns lesions developed, there was an absence of any sequelae, and a reduction in lost work time as compared with similar reported cases decontaminated with water. Five of these >30 cases involved HF exposure circumstances that were potentially life-threatening because of the HF concentration and the exposed body surface area. In these cases, rapid relief of pain was noted, not from any analgesic effect of Hexafluorine®, but most likely from inactivation of the HF, which prevented further tissue damage.

Some experimental studies with rats [28] have challenged Hexafluorine® efficacy, but only when the HF had been left in contact with the skin long enough to result in tissue cellular death before Hexafluorine® was applied. As Hexafluorine® is a decontamination solution and not a treatment for already establish necrotic HF burns, these results are not surprising. In contrast, in two studies, one an *ex vivo* rabbit cornea study using an Eveit® model combined with a powerful OCT imaging technique, Rihawi et al. [58] and Spöler et al. [59] showed that Hexafluorine® was a more efficacious HF decontamination solution than either water or CaG ocular drops.

Water has a primary mechanical washing effect with an added minor diluting effect. As Hexafluorine® is provided as a solution in sterile water, these same washing and diluting effects are also present. However, as water has no chemical effect on HF, it apparently may leave a residue of fluoride ion on the skin in a sufficient quantity to explain the observed secondary necrosis.

Topical CaG has the properties of chelating free fluoride ions, thereby decreasing their concentration in the injured tissue. It can also provide a source of extracellular calcium, which can change the balance between extracellular and intracellular calcium. In the study presented here, a single application of CaG gel following water washing was not sufficient to prevent the delayed development of tissue damage.

Amphoteric Hexafluorine®, in contrast, acts to neutralize the H^+^ ion and chelates the F∼ ion, thereby *actively* acting against both mechanisms of HF cutaneous tissue injury. In the study presented here, no cutaneous tissue injury resulted over 24h of observation from a 20-s application of 70% HF followed by Hexafluorine® washing.

## Conclusion

As demonstrated in the utilized 70% HF-exposed human skin explants *ex vivo,* 70% HF can cause injury to all of the explant skin layers within 5 min after a 20-s application. Decontamination with water followed by a single application of topical 2.5% CaG gel delayed the onset of the skin injury and mitigated the severity of the damage, but did *not* prevent injury in this experimental model. In contrast, when the HF-exposed skin explants were washed with Hexafluorine®, no tissue injury developed during 24-h observation. Thus, in this experimental model, decontamination with Hexafluorine® completely prevented 70% HF cutaneous tissue injury. Hexafluorine® deserves consideration for further study and clinical application.
