# Corrigendum

**DOI:** 10.3109/15569527.2011.613608

**Published:** 2012-02-29

The publisher would like to apologise on behalf of the authors. Since publication of:

Part 2. Comparison of emergency washing solutions in 70% hydrofluoric acid-burned human skin in an established ex vivo explants model François Burgher, Laurence Mathieu, Elian Lati, Philippe Gasser, Laurent Peno-Mazzarino, Joël Blomet, Alan H. Hall, and Howard I. Maibach in Cutaneous and Ocular Toxicology, 2011;30(2):108115, the authors have informed us of the following errors:

In the Methods section for Group F, the times shown in Table 2 are for the actual duration of 70% HF exposure before the 70% HF saturated filter paper was removed and samples were obtained for histological observation. This has effects on various portions of the rest of the manuscript including some Figure Legends, as follows:

In the Methods section,

under “Application of products and washing solutions”, second paragraph, the first sentence should read: “For group F, HF was left in contact with the skin for various times (Table 2) and then the filter paper discs were removed.” Table 2 seems to be correct as published.

under “Histology sampling”, the last sentence of the second paragraph should read: “Then, two explants were sampled and fixed in the same manner with removal of the saturated patches at each of the sampling times: 5, 10, 15, 30 min, 1, 2, and 4h, and 24 h following HF exposure.”

In the Results section,

the second paragraph should read: “Group F (explants exposed to 70% HF for various times (Table 2) on filter paper discs...” the third paragraph should read: “After 20 sec of contact, no deterioration of the structures of either the epidermis or dermis was observed. At 5 min after exposure, very clear cellular deterioration was observed in the epidermis and papillary and reticular dermis. Lesions clearly increased at 10, 15, 30 min and 1 hr after exposure, with the marked appearance of coagulation necrosis including acidophilic cytoplasm and pyknotic nuclei throughout all the skin layers. At 24 h after exposure, the epidermis presented completely necrotic structures. The lesions were less intense in the papillary and reticular dermis (Figure 1A and 1B).” [See errata in Figure 1A & 1B legends below.]

In the Discussion section,

in the third paragraph, the third sentence should read: “By 5 minutes after 70% HF exposure, all the epidermal and dermal layers of the skin were damaged, but not totally and irreversibly destroyed.”

in the sixth paragraph, the third through sixth sentences should read: “Without decontamination washing, the first cellular alterations were noticeable by 1 min after exposure. The reticular dermis was reached and injured by 5 min after exposure. At 5 min after exposure, a massive attack of all the cutaneous layers was already present. The lesions then remained stable after exposures between 10 min and 4 h. At 24 hours after exposure, the epidermis was totally necrosis while the dermis was clearly altered (appearance of coagulation necrosis including pyknotic nuclei and totally acid cytoplasm).”

In the Conclusion,

the first sentence should read: “As demonstrated in the utilized 70% HF-exposed human skin explants ex vivo, 70% HF can cause injury to all of the explant skin layers within 5 min after application.”

The Figure Legend for Figure 1 should read: “Figure 1: (A) and (B) Exposed 70% HF nonwashed explants, aspect at 24 h after exposure. Epidermis (A) and dermis (B). At 24 h after exposure, the epidermis...”

The Table Legend for Table 3, in the footnotes should read: “In the FWCaG and FHexa treatments groups, 70% saturated filter paper discs were left in contact for 20 sec and then removed.”

